# Low Risk of Fracture With End-Range Movements of the Hip in People With Low Bone Mineral Density: A Narrative Review

**DOI:** 10.1177/23337214211052398

**Published:** 2021-11-19

**Authors:** Christina Ziebart, Caitlin McArthur, PT, Judi Laprade, PT

**Affiliations:** 1 Health and Rehabilitation Sciences, 6221 Western University, 1201 Western Rd., London, ON N6A 3K7, Canada; 2 School of Physiotherapy, 3688 Dalhousie University, Halifax, NS, Canada; 3 Division of Anatomy, 7938 University of Toronto, Toronto, ON, Canada

**Keywords:** osteoporosis, risk, shoulder, hip, end-range maneuverers, fractures, stretches

## Abstract

**Background:** The risks of end-range movements for people with osteoporosis, specifically at the hips and shoulder, are not well understood. **Objectives:** To synthesize literature on the safety of stretching for people with osteoporosis by searching: 1) biomechanical literature to determine how much force results from an end-range maneuverer and is required to fracture joint components (focusing on the hip joint) and 2) clinical literature to describe techniques used, populations studied, effects, and reported adverse events. **Methods:** We conducted two separate search strategies in PubMed, EMBASE, and Scopus (1955–2020). **Results:** 16 articles described either biomechanical or clinical effects of passive and active end ranges of the hip joint. The largest load in the hip, described in the literature was in a crescent lunge during yoga. The moment produced in a crescent lunge is much smaller than that of the tensile strength of osteoporotic bone, suggesting the crescent lunge movement could be considered safe. Clinically, no adverse events were reported in exercise, stretching or yoga interventions. **Conclusion:** This review found no evidence that end range movements of the hip are unsafe, but there is little evidence. No studies were identified that explored the risk of humeral fracture during end range stretches.

## Introduction

Osteoporosis is a disease characterized by low bone mass, a deterioration of bone tissue, and a subsequent increased risk for fracture (Prior et al., 2015). The prevalence of osteoporosis increases with age (Prior et al., 2015); however, some women can be diagnosed with osteoporosis perimenopause, as early as in their fifth or sixth decade (Compston et al., 2009; Ismail et al., 2000; Kanis et al., 1994). Conservative management of osteoporosis includes exercise, and recommendations are provided on frequency, intensity, and type of exercise (Giangregorio et al., 2014). However, little information is provided on safety of long lever end-range movements (Jorge Cardoso et al., 2017) like active or passive stretching with osteoporosis.

Stretches performed either by the person themselves or by a practitioner are a common prescription for adults that spend a long time in sedentary positions, or for those presenting with hip pain (Cibulka et al., 2017), and shoulder pain.(Rosa et al., 2017) Sometimes these stretches can be done without regard of a person’s bone integrity and whether or not they have osteoporosis. For adults without osteoporosis, practices such as yoga can be safe and provide relief to muscle tightness that is often associated with a sedentary lifestyle, reducing tightness around the hips and shoulders, or for recovery after activity (Henchoz et al., 2010; Henchoz & So, 2008). However, there are limitations on the movements that may be considered safe. For example, end-range spinal flexion and rotation movements should be limited in any program for people with osteoporosis (McArthur et al., 2016; Sinaki, 2013). End-range spinal flexion and rotation movements are well-disseminated as risky movements in the Too Fit to Fracture exercise recommendations for people with osteoporosis (Giangregorio et al., 2014, 2015). However, there are no guidelines on stretching in terms of dose or potential risks associated with end-range stretching.

Unfortunately, the risks of end-range movements for people with osteoporosis, specifically at the hips and shoulder, are not understood by health care professionals, or patients, and end-range stretches may be advised in clinical practice. Beyond yoga, few studies have assessed the risk of passive, or assisted range of motion activities, particularly in the long bone joints like the hip, in people with osteoporosis. Although a few studies (Giangregorio et al., 2014, 2015; Sinaki, 2013) have suggested there is risk of end-range maneuverers for people with osteoporosis, the consequence of these movements are not well understood. Bone strength is influenced by the material properties, and structural distribution, which is determined by cross-sectional moment of inertia (Beck et al., 2001). The further from the center of mass, the greater the risk of fracture, especially with people with compromised bone strength (Beck et al., 2001). This suggests that long-lever end-range maneuvers may not be recommended for people with compromised bone mineral density.

Therefore, the purpose of this narrative review is to synthesize the literature of what is known about active and passive end-range movements of the hip and shoulder joints, and their safety and applicability for people with osteoporosis. We sought to achieve the purpose by answering the following research questions through a narrative review of the biomechanical and clinical literature:

(1) Biomechanical literature: A) How much force results from a long-lever end-range maneuvers of the long bone joints (focusing on the hip and/or shoulder)? B) How much force is required to fracture a joint with long-lever end-range maneuvers (focusing on the hip and/or shoulder)?

(2) Clinical literature: A) What long-lever end-range maneuvers of the hip and shoulder have been described in the literature for older adults? B) What is the population that the maneuvers been applied with? C) What are the effects of long-lever end-range maneuvers of the hip and shoulder for people with osteoporosis? D) What adverse events have been reported with long-lever end-range maneuvers of the hip and shoulder for people with osteoporosis?

## Methods

### Search Strategy

We conducted a narrative review with two separate search strategies. We specifically focused on studies describing long-lever end-range maneuvers, including stretches of the hip and shoulder regions performed by a therapist or by the individual. Both search strategies were conducted in PubMed, EMBASE, and Scopus between 1955 and 2020. For both searches we excluded non-English reports, books, and overlapping articles between citations.

### Search Strategy: Biomechanical Literature

First, we searched for biomechanical studies that described the force produced either by self or by the clinician by long-lever end-range maneuvers or the force required for a joint to fail from a long-lever end-range maneuvers.

Inclusion criteria were

• Population: individuals or cadavers with compromised bone mineral density.
• Intervention: end-range maneuvers.
• Control: individuals or cadavers without compromised bone mineral density.
• Outcome: force or failure threshold.

Studies were excluded if they were non-English, books or were included in the other search.

We used the key terms: stretch AND hip OR femur OR shoulder OR humerus OR long-lever OR end-range mobilization AND force OR failure threshold.

### Search Strategy: Clinical Literature

Second, we searched for case studies, randomized controlled trials, and systematic reviews describing the effect of, and adverse events associated with long-lever end-range maneuvers for older adults. We chose adults over the age of 55 because this is when they might start to experience complications with reduced bone mineral density.

Inclusion criteria were

• Population: adults over the age of 55.
• Intervention: long lever end range movements such as stretching, yoga, or exercises.
• Outcome: adverse events.

Studies were excluded if they were non-English, books or were included in the other search.

We used the key words: mobilization OR end-range OR stretch(es)(ing), AND osteoporosis.

## Results

A total of 16 articles were identified. Although the intention of this article, and the search strategy were to identify the force created and the effect of active and passive stretching of the hip and shoulder, no articles were found related to the shoulder, making it only possible to provide conclusions about the hip joint.

### Biomechanical Studies

#### (A) How Much Force Results from Long-Lever End-Range Maneuvers?

Eight articles were identified. Five articles discussed the biomechanical demand of long-lever end range maneuvers, particularly active stretching of the hip (Chou et al., 2005; Finley et al., 2001; Omkar et al., 2011; Simoneau et al., 2000; Wang et al., 2013; Westwell et al., 2006). Many of the studies were discussed in the context of yoga for older adults. One study looked at the forces in the hip joints in a variety of standing yoga positions (Wang et al., 2013). The highest load at the hip was generated in a crescent lunge, a movement where the person lunges deeply and leans forward, which was 1.3 Nm/kg. One study mathematically represented the moments around the joints in a “sun salutation” flow, which is a series of end-range spinal flexion and extension both in standing and in prone. The greatest load in the joints explored (wrist, elbow, shoulder, hip, knee, and ankle) were in the hips, specifically during high end-range standing hip flexion (−0.06% body weight), and end-range standing hip extension (−0.085% body weight) (Omkar et al., 2011) (Table 1). In a comparison of studies looking at the moments about the hip during a “sun salutation” the peak load is 0.174% body weight and height normalized. The same article suggests that during daily activities the peak joint moment at the hip is 0.416% of body weight normalized for height (Omkar et al., 2011). These values are presented as a load relative to body weight and height. When looking at load alone, one study reported the moment about the hip during a narrow stance squat is reported as 628 Nm (Escamilla et al., 2001) (Table 1). The force resulting from end range movements is less than the force generated in daily activities.

**Table 1.** The Reported Forces Produced During End-Range Long Lever Maneuvers.

Authors	Participant	Administrator	Protocol	Measurement Apparatus	Location of Applied Force	Mechanical Properties (Mean)
Li & Aspden (1997)	17 osteoporotic femoral heads	Machine tested	Stiffness measured through compression	Compression test using Instron 5564 material testing machine	Femoral head	Stiffness = 247 MPa
						Energy absorbed to yield = 16.3 kJm^−3^
						Material density = 1.6 gcm^−3^
Dickenson et al. (1981)	11 deceased women with femoral neck fractures	Machine tested	Stress–strain measures	Hydraulic servo-controlled testing machine, extensometer	Femoral head	Modulus of elasticity = 11 554 (4169) MNm^−2^
						Ultimate tensile strength= 95.1 (33.8) MNm^−2^
						Plastic energy Absorbed = 0.403 (0.337) MJm^−2^
						Elastic energy Absorbed = 0.274 (0.125) MJm^−2^
						Cavity Area = 27.0 (10.6)%*
						Mineral Content = 65.6 (2.046)%**
						Yield Stress = 75.8 (28.5) MNm^−2^
Trabelsi et al. (2011)	12 femur pairs from deceased males (4) and females (2)	Machine tested	—	qCT-scans (lightspeed VCT, GE healthcare, Waukesha, WI; USA)	Femoral head	Mean stiffness = 1,311 ± 330 N/m
				Servo-electric testing machine (Zwick 010, Zwick GmbH & Co. KG, Germany)		
Schileo et al. (2007)	4 pairs of male cadaveric femurs	Machine tested	Stress–strain measures	Material-testing machine (Mod. 8502, Instron, Canton, MA, USA)	Femoral head	Strain = −1,2001,000 με
Helgason et al. (2008)	1 male femur	Machine tested	Stress–strain measures	Material testing machine (model 8502, Instron, Canton, MA, USA)	Femoral head	Strain = −1,0001,000 με
Bessho et al. (2007)	11 femurs from deceased males (5) and females (6)	Machine tested	Quasi-static compression	Aquilion super 4, Toshiba Medical systems Co., Tokyo, Japan, 120 kVp, 75 mAs, 512 × 512 matrix	Femoral head	Fracture yield load = 3,000–6,000 N
						Fracture load = 4,000–7,000 N
Cristofolini et al. (1996)	4 femurs	Machine tested	Torsional tests	Instron 8502 materials testing machine (Instron ltd. UK)	Femoral head	Mean stiffness = 1,360 N/m
						Antero-posterior bending stiffness = 2,500 N/mm
						Latero-medial bending stiffness = 2,200 N/mm
						Torsional Stiffness = 9 Nm/deg
Papini et al. (2007)	25 femurs from deceased males (5) and females (20)	Machine tested	Axial compression, bending and torsion	Instron 8874 machine (Instron, Canton, MA, USA)	Femoral head	Mean torsional Stiffness = 267 ± 111 Nm/rad
						Axial rigidity = 319 ± 118 kN
						Torsional rigidity = 113±51.2 Nm^2^/rad
						Mean axial Stiffness = 757 ± 264 N/m

N, newtons; S, student; * = *SD*, CI = 95% confidence interval *as a percentage of the cross-sectional are of the specimen **as a percentage of the density of calcite

#### (B) How Much Force is Required to Fracture Hip Components with End-Range Maneuvers?

Several articles (Angin & Erden, 2009; Dickenson et al., 1981; Ott, 1993) were identified discussing the amount of force required to fracture hip components in people with osteoporosis. One study compared the forces required to fracture an osteoporotic femoral bone in vitro compared to a healthy femoral bone (Dickenson et al., 1981). The mean ultimate tensile strength for an osteoporotic bone was 95.1 MN/m^2^ and a mean yield stress of 75.8 MN/m^2^, compared to the healthy subjects where the mean ultimate tensile strength of 117.0 MN/m^2^, and a mean yield stress of 80.8 MN/m^2^ (18) (Table 1). One suggested cause for the lower failure force in people with osteoporosis is due to the increased cavity sizes within the bone due to the decrease in bone mineral density (Dickenson et al., 1981). The same group measured the cavity area as a percentage of density of calcite and found that in people with osteoporosis their cavity percentage was 27%, whereas in health people the cavity percentage was 64%, suggesting that diminished bone density in persons with osteoporosis, leads to an increased fracture risk (Dickenson et al., 1981) (Table 1). However, the reason a person may fracture from a long-lever end-range maneuver may be more complicated than the force applied and the bone density. Other material properties of the bone are likely also contributing to the risk of fracture. It has been noted that osteoporotic bone has a stiffness of 247 MPa within the femoral bone, compared to normal bone, which has a stiffness of 310 MPa (Li & Aspden, 1997). The yield strength is also decreased in osteoporotic bone (2.5 MPa), compared to normal bone (3.3 MPa) (Li & Aspden, 1997). Osteoporotic bone has a much lower ability to absorb energy at 16.3 kJ/m^3^, compared to normal bone at 21.8 kJ/m^3^ (Table 1) (Li & Aspden, 1997). One review article acknowledged that as people age their bone density decreases, increasing the risk of fractures, but, in contrast, the authors found that a 60-year-old woman with a bone density 2 *SD* below normal has a 93% risk of not fracturing, without an explanation as to why (Ott, 1993). It is likely that the person has not yet fractured but is at an increased risk of fracture as they continue to age (Hui et al., 1988). No studies looked at the loads or moments at the shoulder joint, or the force generated from a shear load, which would be a more likely load going through the hip during a long-lever end-range movement.

Using data presented from the literature, we can mathematically assess whether movements like the crescent lunge exceed the strength of osteoporotic bone and suggest a risk of fracture. Consider the moment generated in a crescent lunge position, where the person is in a position of lunging and leaning forward (approximately 1.3 Nm/kg). This was the highest moment reported in the literature, in comparison to the mean tensile strength of 95.1 MN/m^2^ in osteoporotic bone. When applying a conversion to the units, the moment is much smaller than that of the tensile strength per meter squared (see Appendix 1 for the conversion). The moment produced in a crescent lunge is much smaller than that of the tensile strength of osteoporotic bone, suggesting the crescent lunge movement could be considered safe. Importantly, the biomechanical evidence surrounding the safety of end-range movements of the hip is limited and non-existent for the shoulder.

### Clinical Studies

#### (C) What are the Effects of Long-Lever End-Range Maneuvers for People with Osteoporosis?

Table 2 provides a summary of the interventions. Five of the studies focused on stretching programs (Angin & Erden, 2009; Burke et al., 2012; Preisinger et al., 1996; Sherrington, Lord, & Herbert, 2003, 2004). An example of the stretches from one study was to have the participant laying supine while the hip is at 90° flexion. Three studies had the participants perform yoga (Lee et al., 2019; Sinaki, 2013; Tüzün et al., 2010). For example, participants were provided with a 1 h guided hatha yoga practice twice per week for 12 weeks.

**Table 2.** Description of Clinical Studies Using End-Range Long Lever Protocols with People with Osteoporosis.

Authors	Study Design	Population	Long Bone End-Range Intervention	Other Intervention Components	Number of Sessions, Duration of Treatment
Angin & Erden (2009)	Cohort study	• 33 women aged 46-67 diagnosed with osteoporosis	• Exercise program stretching hips, hamstrings, lumber extensors, pectoral stretching, and stretching the vertebral column	• Respiratory exercises, posture exercise, strengthening in supine, standing exercise, and balance exercises	One hour three times a week for 21 weeks
Burke et al. (2012)	RCT	• 50 women over the age of 65 with osteoporosis	• Stretching group	• Balance, muscle strength, and stretching	Maintain static stretching for 1 minute and repeat three times
			• Focusing on major muscle groups of the legs	• Focused on lower extremity	
Lee et al. (2019)	Retrospective cohort	• 89 patients that experienced pain with yoga	• Patients that performed yoga	• None	None
Preisinger et al. (1996)	RCT	• 92 participants Caucasian with moderate idiopathic mid or low back complaints	• Moderate intensity stretching exercises	• Exercises towards improving posture, motor control, coordination, and mechanical efficiency during daily living	20 supervised sessions followed by 3 times weekly for 4 years
		• Without signs of nerve root compression for at least 1 year			
		• Had been postmenopausal for at least 1 year, did not suffer from any disease other than osteoporosis			
		• Were non-smokers and were not taking drugs affecting bone metabolism			
		• Were reported to lead a sedentary lifestyle during the previous 1 years			
		• Women aged 45–75			
Sinaki (2013)	Case series	• 3 healthy people with low bone mass and yoga-induced pain or fracture	• Yoga	• None	None
Sherrington et al. (2004)	RCT	• 120 adults aged 57–95	• The non-weight-bearing group carried out all of the exercises supine including: hip abduction, hip flexion, end of range knee extension, ankle dorsi flexion, and plantar flexion	• The weight-bearing group did the exercises in a weight bearing position including: sit-to-stand, lateral step-up, forward step-up and over, forward foot taps, and a stepping grid	Assessed at baseline, 1 month and 4 months
		• Excluded if unable to complete assessments or the home exercise program			
		• Weight-bearing exercise group *n* = 40			
		• Non-Weight-bearing exercise group *n* = 40			
		• Control group *n* = 40			
Sherrington et al. (2003)	RCT	• 80 older adults in a hospital inpatient rehabilitation ward after a fall-related hip fracture	• The non-weight-bearing group carried out all of the exercises supine including: hip abduction, hip flexion, end of range knee extension, ankle dorsi flexion, and plantar flexion	• The weight-bearing group did the exercises in a weight bearing position including: sit-to-stand, lateral step-up, forward step-up and over, forward foot taps, and a stepping grid	Physiotherapist led exercise program on each weekday for 2-weeks
		• Participants were excluded if they were under age 60, unable to complete the assessments or exercise program			
		• Non-weight-bearing group *n* = 39			
		• Weight-bearing group *n* = 41			
Tüzün et al. (2010)		• 25 postmenopausal osteoporotic women over the age of 55	• Yoga group of 13 people that received yoga intervention 1 h twice a week for 12 weeks. Hatha yoga	• Exercise group of 13 people that performed classic osteoporosis exercises for 1 h twice a week for 12 weeks. Strengthening of the abdomen, back, quadriceps, hamstrings, balance, and posture	1 h twice a week for 12 weeks

Table 3 provides a summary of the outcomes measured, and the results of those outcomes related to loaded hip flexion for people with osteoporosis.

**Table 3.** Reported outcomes of clinical studies using long bone end-range maneuvers with people with osteoporosis.

Reported Outcomes of Clinical Studies Using End-Range Long Lever Therapies			
Authors	Outcomes measured	Outcome measures used	Results
Angin & Erden, (2009)	• BMD	• DXA t-score	• Improvement in t-score in osteoporosis pre (−2.7) and post (−2.4) exercise (*p* = 0.006)
	• Pain	• VAS pain scale	• Improvement in VAS pain score at rest (from 3.7 to 1.0, *p* < 0.001) and during movement (from 5.1 to 2.3, *p* < 0.001)
	• Quality of life	• QUALEFFO-41	• Improvement in quality of life specifically in social activities (from 63.6 to 40.2, *p* < 0.001) and general health (from 51 to 33.9, *p*<0.001)
Burke et al. (2012)	• Hamstring length	• Strength assessed using a dynamometer	• Significant difference between groups hamstring shortening degree, with the control group having the highest degree, and stretching group the lowest degree *p* < 0.05
	• Isometric strength	• Length measured by goniometry	• Ankle flexion and knee extension isometric strength was the highest in the strengthening group, *p* = 0.006
			• Knee flexion isometric strength was the highest in the stretching group *p* = 0.002
(Lee et al., 2019)	• Pain	• Soft tissue injury	• Myofascial pain, and rotator cuff injury were the most common soft tissue injuries
		• Axial non-bony injury	• Degenerative joint disease, radiculopathy, and facet arthroplasty were the most common axial non-bony injuries
		• Bony injury	• Kyphoscoliosis, spondylolisthesis, and compression fractures were the most common bony injuries
Preisinger et al. (1996)	• Osteoporotic fractures	• X-ray	• 44% of participants completed 3 times per week of 20 minutes of exercise
	• BMD, BMC	• Single photon absorptiometry	• 33 participants had had osteoporotic vertebral deformities or a peripheral fragility fracture
	• Pain	• Questionnaires	• There was significant bone loss in the non-compliant and control groups
	• Physical activity		• Back pain decreased significant in the compliant group
			• No changes in physical activity
Sinaki (2013)	• Pain	• Pain	• New pain and fractures were seen in patients that did not experience pain before starting a yoga program
Sherrington et al. (2004)	• Muscle strength	• Maximum voluntary strength of knee extensor Spring gauge for knee extension	• Strength significantly improved in the weight-bearing group for knee extension in the affected and non-affected leg at the 4 months follow up
	• Balance	• Hip abductor and hip flexor muscle strength using a hand-held dynamometer	• No significant strength, balance, gait, or self-reported outcomes for the non-weight-bearing group
	• Gait	• Lateral step-up agility	
	• Functional performance	• Postural sway	
	• Fall risk	• Functional reach	
	• Quality of sleep	• Step test	
	• Pain	• 6 m walk	
	• Self-reported mobility	• Physical performance and mobility examination (PPME)	
Sherrington et al. (2003)	• Strength	• Maximum voluntary strength of knee extensor Spring gauge for knee extension	• No difference was found between group in the change from initial final test performance for the domains of strength (F = 1.67, *p* = 0.14) balance (F = 1.21, *p* = 0.31), or functional performance (F = 0.42, *p* = 0.74)
	• Balance	• Hip abductor and hip flexor muscle strength using a hand-held dynamometer	
	• Gait	• Lateral step-up agility	
	• Functional performance	• Postural sway	
		• Functional reach	
		• Step test	
		• 6 m walk	
		• Maximal vertical force measured on a force plate	
		• Physical performance and mobility examination (PPME)	
Tüzün et al. (2010)	• Balance	• One leg stance	• One leg stance statistically improved pre and post yoga session for the left and right foot (*p* = 0.012, *p* = 0.027)
	• Quality of life	• QUALEFFO	• Total quality of life score statistically improved pre and post yoga *p* = 0.002
Tsauo et al. (2005)	• Hip range of motion	• Goniometer	• ROM, muscle strength, and walking speed did not differ between groups
	• Muscle strength	• Strength of the hip and knee with a hand-held dynamometer	• HHS (from 79.3 to 90.1) and QOL did improve in the PT group (*p* < 0.05)
	• Quality of life	• Harrison hip score	
	• Pain	• HRQOL	
	• Walking speed	• Walking velocity	

Several studies performed stretching-only interventions of the whole body (these were not multi-modal interventions), including that of the hips and shoulders, for people with osteoporosis and found that postural control was improved (Angin & Erden, 2009; Burke et al., 2012; Preisinger et al., 1996), may contribute to prevention of hip fractures (Tüzün et al., 2010), and reduced falls risk, likely due to the improved balance. Fractures were not an outcome of three studies (Angin & Erden, 2009; Burke et al., 2012; Tüzün et al., 2010), and no adverse events were reported (Tables 2 and 3), however, it is difficult to determine whether the benefits and risk of the programs were from stretching alone, or from other components of the exercise programs.

#### D) What Adverse Events Have Been Reported with Long-Lever End-Range Maneuvers?

One study conducted a case series of adverse events after yoga (Sinaki, 2013). The cases included women both younger (61 years of age) and older (87 and 70 years of age), with varying progression of osteoporosis and co-morbid conditions that all experienced pain in the hip with end-range maneuvers during yoga. A review article also found a variety of adverse events associated with yoga in patients with osteoporosis, which included myofascial pain due to overuse, rotator cuff injury, among other injuries not related to the hip or shoulder, and therefore not a focus of this review (Lee et al., 2019). A systematic review broadly looked at the relative risk of any fracture after an exercise intervention, which included components of long-lever end-range movements in patients with osteoporosis and found no difference between the intervention and control (Lock et al., 2006). Other studies, described in section C, have looked at adverse events in exercise interventions in people with osteoporosis (Chilibeck et al., 2011; Sherrington et al., 2003, 2004), and none of the adverse events that result in fractures were associated with stretching (Tables 2 and 3).

## Discussion

This narrative review was conducted to examine the safety and applicability of end-range hip and shoulder loads for people with osteoporosis through two approaches: 1) a biomechanical approach to determine the forces applied during long lever movements of the hip and shoulder; and 2) a clinical perspective to determine the effects and adverse events that may be associated with hip and shoulder stretching. However, no information was found on the shoulder. Although bone mineral density is decreased and people with osteoporosis require a smaller load to fracture, there have been few reported adverse events in exercise trials involving people with osteoporosis, and no fractures associated with these active and passive end-range of motion of the hip. Further, few studies have described the load at the hip or described how much force is required to fracture these areas specifically, so it is challenging to discern the risk associated with these movements.

### Forces Applied During Long-Lever End-Range Maneuvers Compared with Forces Required to Fracture Components Associated with the Long-Lever End-Range Maneuvers

The studies quantifying the load associated with active and passive end-range of motion in the hip were in older adults participating in yoga (Chou et al., 2005; Finley et al., 2001; Omkar et al., 2011; Simoneau et al., 2000; Wang et al., 2013; Westwell et al., 2006). The greatest loads were determined to be in end-range movements but the loads did not appear to exceed that of daily activities (Escamilla et al., 2001; Holzbaur et al., 2007). When compared to the amount of load required to fracture hip components, it appears that the load would not be sufficient to fracture an osteoporotic hip. Factors such as stiffness, tensile strength, yield load, and microarchitecture of the bone (Dickenson et al., 1981) will contribute to whether someone will fracture from these end-range movements of the hip. However, it is important to recognize that these are theoretical observations from a variety of studies, with no studies specifically identifying loads during end-range active or passive stretching of the hip. It would be unethical to directly observe the threshold to fracture for patients with osteoporosis, but a more sophisticated mathematical model, or cadaveric examination may provide insight into the true risk of end-range stretching in people with compromised bone mineral density, looking at multiple joints including the hip, shoulder, wrist and vertebrae.

### Effects of Interventions That Include Long-Lever End-Range Maneuvers Compared with Adverse Events

None of the studies used in this narrative review reported fracture-related adverse events for active or passive end-range movement in the hip, in people with osteoporosis, with all studies being participant-initiated stretches, not therapist or practitioner assisted stretches. Although none of the studies explored in this narrative review reported an increased risk of hip fracture, there were adverse events related to soft tissues such as myofascial pain and rotator cuff injury secondary to stretching interventions for people with osteoporosis. The increased risk of pain may be due to a stiffening of the connective tissue surrounding the joints, that occurs with aging (Parry et al., 1978; Woo et al., 1986), and progressing into inappropriate ranges of motion too quickly. No studies have looked at the effect of a variety of long-lever end-range maneuvers, or the use of practitioner-assisted stretching, and more work should be done to examine the effects of end-range stretching passively and actively, in standing and in supine for people with osteoporosis before a true understanding of the risk of stretching is understood in people with osteoporosis. More work in this area could provide practitioners and researchers with the peace of mind that end-range stretching is safe for people with osteoporosis, or know what parameters to work within to reduce the risk of fracturing with end-range stretching.

### Suggestions for Clinical Practice

Although no explicit adverse events related to fracturing were observed for end-range long-lever maneuvers in people with osteoporosis, it is still recommended to adhere to the osteoporosis-specific exercise recommendations, Too Fit to Fracture (Giangregorio et al., 2014, 2015) and avoid excessive or loaded end-range movements to reduce the risk of fracturing. The current literature supports self-generated lunges and standing hip flexion are safe for people with osteoporosis. There may be additional benefits for engaging in stretching exercises for people with osteoporosis, such that improving range of motion can improve mobility and help reduce the risk of falling. As well, activities like yoga have benefit beyond mobility and can be beneficial for mental health and improving posture. Hyperkyphosis is a common consequence of people with osteoporosis, which can also limit shoulder range of motion, potentially contributing to adverse events associated with shoulder stretching, but this is not clear based on the literature and more clinical studies need to be conducted. Finally, Pilates is a popular mode of exercise for a lot of older adults, but there is very little information on the safety of Pilates for people with osteoporosis. There is generally more end-range spinal flexion and twisting in Pilates than yoga, suggesting many postures would not adhere to the Too Fit to Fracture guidelines and should be avoided.

Benefits of range of motion activities should be evaluated to determine whether they will accomplish the goals, or whether other strategies might be more appropriate. Decisions around end-range movements should consider the individual, their previous level of activity and flexibility, their goals for treatment, and clinician competence. The practitioner should feel comfortable providing advice to people with osteoporosis about exercise and have a competent understanding of general limitations for activity with people with osteoporosis, considering the individual.

## Conclusion

In conclusion, this narrative review summarized the literature on end-range active and passive range of motion activities for the hip with biomechanical and clinical considerations for people with osteoporosis. This review found no evidence that end range movements of the hip are unsafe, but there is little evidence to provide firm guidance for practitioner assisted stretches in standing or in supine. No studies were identified that explored the risk of humeral fracture during end range stretches.

**Acknowledgements**

Christina Ziebart is supported by the CIHR doctoral award. We would also like to acknowledge of Ms. Rachel Kiefte for her support with Appendix 1.

## Appendix 1

To be able to compare 95.1 MN/m^2^ and 1.3 Nm/kg, we’ll apply a unit conversion.

First, we can note that 1 Newton-meter (Nm) is algebraically equivalent to 1 Joule (J), so 1.3 Nm/kg = 1.3 J/kg.

For ease of calculation, we will also use scientific notation: 95.1 MN/m^2^ = 9.51 × 10^7^ N/m^2^.

Since 1 Pascal (Pa) is equivalent to 1 N/m^2^, 9.51 × 10^7^ N/m^2^ = 9.51 × 10^7^ Pa.

Further, 1 Pa is also equivalent to 1 J/m^3^, so 9.51 × 10^7^ Pa = 9.51 × 10^7^ J/m^3^.

Although 1 m^3^ = 1000 kg wt, bone does not have the same density as water. In fact, 1 m^3^ = 1850 kg of bone (note that this will be lower for osteoporotic bone).

Therefore, 9.51 × 10^7^ J/m^3^ = 9.51 × 10^7^ J/1850 kg = 5.14 × 10^4^ J/kg.

5.14 × 10^4^ J/kg would be the tensile strength of healthy bone, compared to the 1.3 J/kg which is the moment generated in the crescent lunge position, which is much larger. Although osteoporotic bone will have a smaller density, the tensile strength is still significantly larger than the moment created by a crescent lunge, suggesting a low risk for fracture.
